# Medical and Physical Intelligence

**Published:** 1806-10

**Authors:** 


### MEDICAL AND PHYSICAL INTELLIGENCE.

Dr. GIBBES has from a series of experiments shewn, that the Bath waters contain a much greater portion of iron than has hitherto been supposed. He says, that "iron is deposited in three different states by the Bath waters: 1. It tinges the glasses which are made use of for drinking the water at the pumps of a yellow golden colour, which can be scraped off. This portion is what I imagine was united with carbonic acid, and is deposited on the glasses, on the sides and bottom of the baths, in the state of ochre.—2. It forms pyritical incrustations about the reservoirs and channels of the baths: in these the iron is, in its metallic state, united with sulphur.—3. It is deposited in the sand of the bath in black particles, which are attracted by the magnet. Some of these particles appeared in a crystalline form."

---

Dr. BUCHAN, in his answer to Sir John Sinclair's pamphlet on the subject of Athletic Exercises, speaking of the danger of drinking cold liquors when the body is heated by exercise, says that immediate death has not seldom been the consequence of drinking a glass of cold water or beer, after having been heated and fatigued by dancing or any other violent exercise. To those who may inadvertently be guilty of such imprudence, it may be well to know that to swallow immediately a glass of brandy, or a teaspoonfull of laudanum, is the best means of counteracting its baneful consequences.

From the same authority we learn, that many within the doctor's knowledge, who, after having suffered severely from repeated attacks of the gout, have completely eradicated that disorder, by an entire abstinence from fermented liquors of all kinds; and have by the same means recovered a much greater share of health and vigour than could have been expected.

---

The effects of *diæta aquea*, or living wholly on pure water cooled by ice, in alleviating the pain of cancer, and in several cases even of its effecting a complete cure of that painful disease, which are narrated by M. Pauteau, and which have been corroborated by the experience of Mr. Pearson, have, says Dr. Buchan, been unaccountably neglected. Nevertheless, after a few days the desire for solid food entirely subsided, and the stomach appeared completely satisfied when filled with the aqueous fluid.

---

Dr. DENNISON and Dr. SQUIRE will, on Saturday the 4th of October, begin a Course of Lectures on the Theory and Practice of Midwifery, and the Diseases of Women and Children.

Mr. GARDNER will deliver the Introductory Lecture to his Autumnal Course of Chemical Lectures, on Monday evening the 6th of October, 1806, commencing at eight o'clock precisely, at the Paul's Head Tavern, Cateaton Street. The following Lectures will be given at the City Dispensary, Grocers' Hall Court, Poultry, every Wednesday and Friday evenings, at the same hour, where gentlemen may apply for particulars.

---

Dr. HOOPER will commence a Course of Lectures on the Theory and Practice of Physic, the Materia Medica, and Pharmaceutical Chemistry, on Monday, October 13, at his Lecture Room in Cork Street, Burlington Gardens, at eight o'clock in the morning. A Prospectus, with particulars, may be had at the Doctor's house, No. 21, Saville Row.

---

Mr. MOOR, Surgeon-Dentist to Her Royal Highness the Duchess of York, will commence a Course of Lectures on the Structure and Diseases of the Teeth, on October 30, in which will be explained the Complete Practice of the Dentist. Further particulars may be known by applying at his house, No. 6, Palsgrave Place, Temple.

---

Dr. REID will commence his Winter Course of Lectures, on the Theory and Practice of Medicine, on Saturday the 25th of October, at Eight o'Clock in the Evening, at his house, No. 6, Grenville Street, Brunswick Square, where the Course will be continued at Ten o'Clock in the Morning, on Monday, Wednesdays, and Fridays.

---

Dr. HAMILTON, of Halesworth, is about to publish a popular Treatise on the Cause and Prevention of Gout.

---

Mr. SAMUEL YOUNG is about to arrange a Collection of Facts in the form of a Dissertation on the Advantages of the Adhesive Strap, shewing the abuses of the ligature in the stitching of wounds. This work may be expected in the course of the winter.

---

